# Protocol for a factorial randomised controlled trial, embedded within WHiTE 8 COPAL, of an Enhanced Trainee Principal Investigator Package and Additional Digital Nudge to increase recruitment rates

**DOI:** 10.12688/f1000research.19743.1

**Published:** 2019-07-22

**Authors:** Nickil Agni, Caroline Fairhurst, Catriona McDaid, Mike Reed, David Torgerson

**Affiliations:** 1 Department of Health Sciences, University of York, UK, York, YO10 5DD, UK; 2 Department of Orthopaedics, Northumbria Healthcare NHS Trust, Ashington, NE63 9JJ, UK

**Keywords:** Trainee Principal Investigator, TPi, Nudge, Swat, Recruitment, Education

## Abstract

Recruitment remains an issue when conducting randomised controlled trials (RCTs) with a significant proportion of studies failing to reach their target sample size. Studies evaluating interventions to improve recruitment aimed specifically at recruiters to the trial are limited in number. This factorial RCT will evaluate the effectiveness of an educational intervention to trainee principal investigators and a positive reinforcement intervention via an email nudge on increasing recruitment. The targeted recruiters will be in 20 centres nationally recruiting to one large orthopaedic randomised controlled trial, WHiTE 8 COPAL. Centres will be randomised via minimisation to one of four groups. The primary outcome is recruitment rate in the first six months that a centre is actively recruiting, with data being analysed via a Poisson regression model. Results will be presented as adjusted incidence rate ratios with 95% confidence intervals. Secondary outcomes relate to the feasibility and logistics of running the interventions.  We will also collect feedback regarding the educational programme set out for the trainee principal investigators. The study started in August 2018 with the anticipation of the primary objective endpoint by October 2019. The results of this study will be used to inform the design of future RCTs, particularly in orthopaedics in the UK, where the role of Trainee Principal Investigators is now a consistent one across different trials.

**Trial registration:** 11600053, ISRCTN, 20/08/2018; SWAT 67, Northern Ireland Hub for Trials Methodology Research SWAT repository, 01/10/2017.

## Introduction

Randomised controlled trials (RCTs) are considered the gold standard when evaluating the efficacy and effectiveness of health care interventions. Unfortunately, a significant number of well-designed RCTs struggle with the recruitment of participants and subsequently fail to reach their target sample size ^1^.

Several hypothetical and real-life studies on methods to improve participant recruitment to RCTs have been conducted with mixed results, with only a minority targeting recruiters to the trial ^2– 5^. The results of a survey of clinical trials units in the UK concluded that priorities for evaluation included training site staff, methods of communication with patients and incentivising site staff ^6^.

The aim of this real-life study is to assess the effects of targeting healthcare professional recruiters with an educational intervention with or without positive reinforcement on participant recruitment. This study within a trial (SWAT) will test two different methods of enhancing recruitment: introducing an enhanced trainee principal investigators (TPI) package, and personalised email nudges (see *Extended data* ^7^) to healthcare professionals involved in patient recruitment. The SWAT will be implemented in a large, UK, multicentre orthopaedic RCT, the WHiTE 8 COPAL trial. The interventions have both been used in current orthopaedic trials, but their effects on recruitment have previously not been investigated.

## Interventions

This will be a multicentre, 2×2 factorial RCT run between August 2018 and October 2019 with random allocation of the recruiting centre to one of four groups:

Group 1: Enhanced TPI package.
Group 2: Use of a personalised email nudge to each recruiter.
Group 3: Enhanced TPI package and use of a personalised email nudge to each recruiter.
Group 4: Usual practice (neither the enhanced TPI nor personalised email nudge).

Full details of each intervention have been provided as *Extended data* ^7^ and are summarised in Table 1. The consent to participate as a TPI and interventions will be implemented by the White 8 Research Fellow (author N.A.). Consent materials are also available as *Extended data* ^7^.

**Table 1.** Summary of additional activities in each intervention group.

Activity	Usual practice	Enhanced TPI	Email nudge
Identify TPI for the trial	Through local Principal Investigator	Through local Principal Investigator	
Training of TPI regarding how to perform their role once centre is activated for recruitment	Local Principal Investigator TPI Manual	Local Principal Investigator WHiTE 8 Research Fellow via 1:1 telephone induction TPI manual Induction summary presentation	
Training TPI regarding the WHiTE 8 trial and consenting procedures	Local Principal Investigator	Local Principal Investigator WHiTE 8 Research Fellow via 1:1 telephone induction WHiTE 8 consent flow diagram and protocol provided	
Peer-support of TPI		Monthly contact by WHiTE 8 Research Fellow WHiTE 8 Research Fellow can be contacted by TPI as required by SMS/WhatsApp/Email	
Digital information provided to TPI	TPI Manual	Induction agenda TPI manual and new TPI checklist Induction summary presentation WHiTE 8 consent flow diagram and protocol TPI contact information consent form	
Identifying patients for the trial	Trauma meeting	Trauma meeting	
Confirmation of randomisation	Automated email to recruiting centre		Automated email to recruiting centre Additional personalised email to express gratitude and encourage further recruitment within 72 hours of randomisation

TPI, trainee principal investigator

## Sample selection

As in many SWATs, a power calculation was not undertaken as the number of participating sites is fixed and driven by the needs of the host trial. All WHiTE centres planned to be recruiting to the WHiTE 8 trial will be included, except the centre in which N.A. is based. We anticipate a minimum of 20 centres being involved in recruiting. Trial interventions will only be discontinued if the host trial (WHiTE 8 Copal) is discontinued.

## Randomisation

The WHiTE centres will be randomised by minimisation on a rolling basis as sites become activated to one of the four groups to balance key baseline characteristics. Self-reported site feasibility questionnaires completed by the recruitment centres will be used to collect the information required for the minimisation. Minimisation will be based on the following factors:

1. Cluster size (number of intracapsular hip fractures presenting in the previous year, cut at the median <300 or ≥300)
2. High vs Low recruiting centres (<9 or ≥9 per month based on previous RCTs run within WHiTE Cohort)
3. Co-recruitment to WHiTE 5 (yes/no) (Another RCT using the same patient population running at a few of the recruitment sites)

This randomisation will be performed using specialist computer software, MinimPy (Saghaei and Saghaei, 2011). This is an open trial and participating sites, the data analyst nor trial team will be blind to allocation.

## Outcomes

The primary outcome is the total number of patients recruited in the first 6 months from a site opening to recruitment to the WHiTE 8 COPAL trial.

The secondary outcomes are: conversion rate from screened population collected monthly from the central recruitment database (coordinated by the Oxford Clinical Trials Research Unit); and the time taken to implement each intervention from commencing recruitment in each centre.

The trainee’s perspective of their role will be collected through the TPI survey (available as *Extended data* ^7^ at the end of the SWAT in each centre. The Research Fellow will keep a record of the time taken delivering the TPI education intervention and a log of communication for peer-support during the period of the SWAT to inform future implementation.

## Ethical issues

The University of York Health Sciences Ethics Committee has approved this study within a trial. Ethics Approval ID: HSRGC/2018/266/C. Substantive protocol amendments will be sought approval through then university ethics committee.

## Trial registration

This SWAT is registered with ISRCTN ( 11600053) and is embedded in the WHITE 8 Copal trial (ISRCTN 15606075).

This SWAT is also registered to the SWAT repository store as part of the Northern Ireland Hub for Trials Methodology Research ( SWAT 67).

## Data analysis

Analysis will be conducted in STATA v15 on an intention-to-treat basis, including all sites in the group they were originally allocated to regardless of deviations based on non-compliance. Statistical significance will be assessed using logistic regression two-sided statistical tests at the 5% significance level. The trial will be reported to CONSORT guidelines, and a flow diagram will present the progression of sites through the trial.

Baseline data relating to the sites (including the minimisation factors) will be summarised for the four groups as randomised and as analysed to assess whether possible loss-to-follow-up has introduced selection bias. Continuous data will be presented using descriptive statistics (e.g., mean, standard deviation, median, minimum, maximum), while categorical data will be given as counts and percentages. No formal statistical comparison of baseline data will be undertaken between the four groups.

The number of participants recruited per site will be summarised. A Poisson regression model, containing the two interventions (Enhanced TPI and Email Nudge) and the minimisation factors (cluster size, and number recruited per month will be included in their continuous form) will be undertaken. Adjusted incidence rate ratios (IRRs) and associated 95% confidence intervals (CIs) will be obtained from this model. The presence of an interaction between the two interventions will also be tested by including an interaction term in the model. 

Feasibility outcomes, such as the time required to run the education intervention and communication time and methods used for the peer support aspect of the intervention, will be reported descriptively.

A data monitoring committee will not be used as this a trial involving recruiters and patient safety will not be affected by conducting this trial. No formal auditing of trial procedure will take place.

## Discussion

If successful, we would like to show that these can be feasibly implemented in future RCTs with additional benefit of reaching targeted sample sizes within the planned recruitment timeline due to increased recruitment rates.

## Plans for dissemination

Results of this study will be form part of a PhD thesis, published in a peer-reviewed journal, presented at conferences and be shared with recruiting centres and clinical trials units.

## Data availability

### Underlying data

No underlying data are associated with this article.

### Extended data

Open Science Framework: Protocol for a factorial randomised controlled trial, embedded within WHiTE 8 COPAL, of an Enhanced Trainee Principal Investigator Package and Additional Digital Nudge to increase recruitment rates. https://doi.org/10.17605/OSF.IO/FZ4JH ^7^.

This project contains the following extended data:

• Extended SWAT Protocol
• Nudge email 1
• NUDGE MATRIX
• TrainingPackage_V1_2017-03-14 (trainee principal investigator manual)
• Consent for contact
• Enhanced TPi Induction Agenda ver1.0apr18
• New TPI Checklist
• SWAT Participation info ver3apr18
• TPI Induction Presentation
• TPi_Follow_up_Survey

### Reporting guidelines

Open Science Framework: SPIRIT checklist for article ‘Protocol for a factorial randomised controlled trial, embedded within WHiTE 8 COPAL, of an Enhanced Trainee Principal Investigator Package and Additional Digital Nudge to increase recruitment rates’. https://doi.org/10.17605/OSF.IO/FZ4JH ^7^.

Data are available under the terms of the Creative Commons Zero "No rights reserved" data waiver (CC0 1.0 Public domain dedication).
