# Supplementary material for: Protocol for a factorial randomised controlled trial, embedded within WHiTE 8 COPAL, of an Enhanced Trainee Principal Investigator Package and Additional Digital Nudge to increase recruitment rates
Source: F1000Res. 2019 Jul 22;8:1153. [Version 1] doi: 10.12688/f1000research.19743.1 (PMC6733373; doi:10.12688/f1000research.19743.1)
Supplement: Peer review history

# Peer review: Reviewer response for version 1

**DOI:** 10.5256/f1000research.21652.r52279

**Published:** 2019-09-05

**Authors:** Kath Star, Victoria Bell

**Affiliations:** 1 Centre for Healthcare Randomised Trials (CHaRT), Health Services Research Unit, University of Aberdeen, Aberdeen, UK; 2 The Centre for Healthcare Randomised Trials, University of Aberdeen, Aberdeen, UK

This is an interesting and well designed trial and the outcomes will be of interest to many involved in running clinical trials.

 There are a few suggestions/areas for consideration by the authors: 

- The manuscript would benefit from the addition of a description of the role of a TPI should be included (as stated in section 1.3.2 of the extended protocol)
- Have the authors considered additional timepoints for follow up? Recruitment fatigue can often affect long-running studies and it would be interesting to determine whether the intervention has longer-lasting effects. 
- Feasibility and resource use are an important aspect of the study and should be included in the secondary outcomes.
- Have the authors considered performing a formal cost analysis? It would be interesting to understand the cost/benefit of the intervention.
- There are a number of acronyms referring to the studies hosting or associated with EnTraP – WHiTE, WHiTE 8, WHiTE 8 Copal, WHiTE 5 - that we found confusing and detracted from the description of the study. Section 1.3.1 of the extended protocol explains the relationship between these studies well, and it would be useful to include this for the reader to understand the context of the host trial.
- In the extended protocol the study is referred to as EnTraP and it would be beneficial to include this in the article to refer to e.g. ‘the *EnTraP *Research Fellow’.
- Will education and experience of the sites staff be mentioned or evaluated when analysing the results as this can impact on recruitment.

The extended protocol submitted as extended data still has some comments and tracked changes the authors may want to remove before indexing.

We confirm that we have read this submission and believe that we have an appropriate level of expertise to confirm that it is of an acceptable scientific standard.

# Peer review: Reviewer response for version 1

**DOI:** 10.5256/f1000research.21652.r52423

**Published:** 2019-09-03

**Authors:** Mike J. Clarke

**Affiliations:** 1 Centre for Public Health, Queen’s University Belfast, Belfast, UK

Struggling or failing to achieve their target recruitment, and therefore, failing to have adequate statistical and clinical power, is a problems for many clinical trials. It contributes to research waste and slows down our ability to resolve important uncertainties in health and social care.

 This is a well reported protocol for a SWAT (Study Within A Trial) that will be embedded in the WHiTE 8 COPAL randomised trial. The SWAT will investigate ways to increase recruitment in the clinical trial. I think it should be indexed and I wish the authors well with the conduct and analysis of the SWAT. It might be helpful to add a reference to a more general article about SWAT to help readers who would like to find out more (e.g. Education section – Studies Within A Trial (SWAT). (2012) ^1^; or Smith V, Clarke M, Devane D, *et al*. (2013) ^2^; or Treweek S, Bevan S, Bower P, *et al*. (2018) ^3^).

 The methodology study has been registered as SWAT 67 ( go.qub.ac.uk/SWAT-SWAR) and is also registered as ISRCTN11600053. It will be a factorial, cluster randomised trial in which recruiters at each of the 20 sites for WHiTE 8 COPAL will be allocated to one of four groups. The two interventions in the factorial design are the introduction of an enhanced trainee principal investigators' (TPI) package and personalised email nudges, which are sufficiently different to meet the criteria for the good use of a factorial trial design. There is an adequate summary of the interventions in the body of the article. The use of minimisation to allocate the centres to one of the four groups is an appropriate strategy to help achieve balance, especially with such a relatively small number of units being randomised. Apart from my concern below, the minimisation variables seem reasonable. However, might variables 1 (cluster size) and variable 2 (monthly recruitment in previous randomised trials in WHiTE COPAL) be so closely correlated that they will serve as a single variable only? For instance, my presumption would be that the smaller centres (i.e. <300 cases) would have the lower recruitment (i.e. <9/month), and the larger centres would have the higher recruitment.

 My concern is with the primary outcome, which is the number of patients recruited in the first six months after a centre opens. My concern is that there might be such wide variation in these numbers that, with perhaps five centres in each of the four allocation groups (or ten in each of the groups testing each of the two interventions) that underlying differences between the centres might dominate the final results. I realise that cluster size will be used in the minimisation and in the analysis but this seems to be dichotomous around a median of 300 cases in the previous year which, depending on the distribution of the cluster sizes, might be too simplistic a split. For example, if there are three sites with less than 50 cases in the previous year, or three with more than 500 cases, these might highly skew the data if, by chance, all three at the low or at the high end are randomly allocated to one of the interventions. It would be reassuring to know that the centres are not so heterogeneous or, if they are, that a finer level of adjustment than below and above 300 will be used in the analysis.

 In summary, this is an important study. It will provide evidence relevant to other orthopedic trials, that may help future researchers to boost recruitment (if one or both interventions are effective) or to re-direct SWAT to other areas if they are not. Its relevance is also likely to extend beyond clinical trials in this setting, by contributing to the overall evidence base on interventions to boost recruitment, which have been shown to be so lacking in the Cochrane Methodology Review (reference 2 in this manuscript).

I confirm that I have read this submission and believe that I have an appropriate level of expertise to confirm that it is of an acceptable scientific standard.

## References

[1] Education section - Studies Within A Trial (SWAT). *J Evid Based Med*.2012;5(1) : 10.1111/j.1756-5391.2012.01169.x 44-5 10.1111/j.1756-5391.2012.01169.x 23528122

[2] : SWAT 1: what effects do site visits by the principal investigator have on recruitment in a multicentre randomized trial?. *J Evid Based Med*.2013;6(3) : 10.1111/jebm.12049 136-7 10.1111/jebm.12049 24325369

[3] : Trial Forge Guidance 1: what is a Study Within A Trial (SWAT)?. *Trials*.2018;19(1) : 10.1186/s13063-018-2535-5 10.1186/s13063-018-2535-5 29475444PMC5824570
